# Spotlight on Leishmaniasis Research: Insights from the Special Issue “Emerging Topics in Leishmaniasis Research”

**DOI:** 10.3390/tropicalmed9090200

**Published:** 2024-09-02

**Authors:** Sandra Regina Maruyama

**Affiliations:** Department of Clinical Analyses, Toxicology, and Food Science, School of Pharmaceutical Sciences of Ribeirão Preto, University of São Paulo, Ribeirão Preto 14040-903, SP, Brazil; sandrarcm@usp.br

Leishmaniases, caused by dixenous trypanosomatids from the Leishmaniinae subfamily (over 20 *Leishmania* species), manifest in three primary clinical forms: visceral (VL), cutaneous (CL), and mucocutaneous (MCL). VL is the most severe form that affects bone marrow, spleen, and liver, being fatal if untreated or misdiagnosed [1]. All forms present challenges in diagnosis, treatment, and control/prevention, demanding multifaceted and innovative research approaches [2,3], as each host–pathogen combination evokes distinct strategies for parasite establishment, survival, and persistence [4]. Understanding this diversity is crucial for developing vaccines, drugs, and therapeutic interventions.

There are nearly 100 endemic countries where leishmaniasis is reported to cause 0.7–1 million new cases annually. More than 90% of cases of VL reported globally are from seven countries: Brazil, Ethiopia, India, Kenya, Somalia, South Sudan, and Sudan [5,6]. Once confined to tropical and subtropical regions, it is now increasingly being reported in non-endemic areas, particularly in Europe [7], which remains unprepared to handle this emerging threat. The disease has expanded beyond its traditional ecotopes due to ecological disruptions caused by human activities, driven by factors such as migration, environmental changes, war, urbanization, globalization, and immunosuppression [8].

Recent findings have added a new layer of complexity to the understanding of leishmaniasis. A report has confirmed co-infection by a novel species of trypanosomatid (from the *Crithidia* genus) in a refractory relapsed VL patient in Brazil [9], challenging the traditional dichotomy between monoxenous and dixenous trypanosomatids. This discovery, which aligns with more studies indicating the presence of other trypanosomatids in patients diagnosed with leishmaniasis (reviewed by Kaufer et al. [10] and Boucinha et al. [11]), underscores the multifaceted nature of this disease. The persistent spread of leishmaniasis, even in regions previously considered non-endemic, and the emergence of co-infections highlight the urgent need for ongoing vigilance and adaptive strategies to manage this increasingly complex and global threat.

As the first edition of Special Issue “Emerging Topics in Leishmaniasis Research” draws to a close, it is important to reflect on the significant strides made in understanding and managing this complex disease. Recent advancements have been pivotal in enhancing our understanding of the intricate interactions between the parasite, patients, vector sand flies, and natural reservoir hosts. This Special Issue has illuminated several critical areas where knowledge gaps have persisted, bringing to light new molecular participants in parasite/host interactions, innovative diagnostic tools, and insights into therapeutic approaches.

## 1. Vector–Host Dynamics and Epidemiological Perspectives

The study by Khogali et al. (contribution 1) on the infection of *L. donovani* in *Phlebotomus orientalis* sand flies at different microhabitats in an endemic village of Eastern Sudan provides insights into the ecological aspects of *Leishmania* transmission. By examining the vector–host dynamics in varied microhabitats, this research highlights the importance of targeted vector control programs in East Africa. In addition, Cañeda-Guzmán et al. (contribution 5) present a comprehensive entomological survey conducted during a cutaneous leishmaniasis outbreak in Mexico, revealing the prevalence of *L. mexicana* in sand fly populations belonging to four different genera of phlebotomines and its implications for disease control.

Contributing to understand the epidemiological trends and clinical manifestations of cutaneous leishmaniasis in Saudi Arabia, the retrospective study from Al-Dhafiri et al. (contribution 2) provides a detailed overview of metadata from 245 CL patients attended in Al-Ahsa between 2017 and 2023, focusing on its prevalence and offering insights into regional disease patterns.

## 2. Co-Infection Complexities and Diagnostic Innovations

Investigations into co-infections, such as the study by Monteiro et al. (contribution 3) on proinflammatory chemokines in HIV patients with asymptomatic *L. infantum* infection and the review work by Camelo et al. (contribution 7) on the *Schistosoma*-*Leishmania* co-infections, underscore the need for integrated disease management strategies.

One of the key contributions in this issue is the study by Takamiya et al. (contribution 6) on parasite detection in visceral leishmaniasis using dye-based qPCR, which uncovers the co-infection of *L. infantum* with *Crithidia* sp. LVH-60A [12], a novel trypanosomatid species. The study’s use of species-specific primers has demonstrated high accuracy, revealing that these co-infections are more common than previously recognized. This finding challenges the traditional dichotomy between monoxenous and dixenous on trypanosomatid’s parasitic lifestyle and underscores the need for revised diagnostic protocols and therapeutic strategies to address the added complexity in managing leishmaniasis cases.

## 3. Therapeutic Evaluations and Alternative Prophylaxis

Therapeutic improvements are crucial for effective disease management and control programs for zoonoses. Vaz et al. (contribution 4) conducted a clinical trial evaluating the response of domiciled dogs with visceral leishmaniasis treated with miltefosine and allopurinol, which offered valuable insights into potential treatment regimens. Veterinarians and public health officials can benefit greatly from their findings. Complementing this, the systematic review by Santos et al. (contribution 8) on treatment failure and clinical relapses provides a nuanced understanding of the multifactorial aspects influencing therapeutic outcomes, emphasizing the complex nature of Leishmaniasis. Further highlighting these challenges, the study by Ferreira et al. (contribution 10) on in the vitro drug susceptibility of an L. infantum clinical isolate from a pediatric patient after multiple relapses found that the isolate remained sensitive to common antileishmanial drugs. This suggests that the patient’s treatment failures were likely due to immunophysiological factors rather than drug resistance, underscoring the need for deeper exploration into patient-specific conditions.

In addition to therapeutic strategies, an innovative approach to prophylaxis was also explored. The study by Varotto-Boccazzi et al. (contribution 9) investigates the immune response elicited by the rectal administration of L. tarentolae cells in mice. This approach, originally repurposed from an anti-viral vaccine study, reveals a specific, Th1-associated IgG2a response, opening up new perspectives for mucosal vaccination against leishmaniasis.

## 4. Future Directions in Leishmaniasis Research

To continue making strides against this complex disease, future research should focus on several key areas:

I.   Integrated disease management: developing comprehensive strategies that combine prevention, diagnosis, treatment, and vector control to reduce transmission and manage outbreaks effectively.
II.  Vaccine development: prioritizing the creation of safe, cost-effective vaccines that provide long-term immunity against various forms of Leishmaniasis.
III. Molecular and genomic studies: harnessing genomics and proteomics to identify new biomarkers for early diagnosis and potential therapeutic targets, understanding genetic diversity, and mechanisms of drug resistance.
IV. Socio-economic research: investigating the socio-economic determinants of Leishmaniasis to develop public health policies that reduce stigma and improve access to healthcare services.
V.  Environmental and climate impact: studying the effects of climate change and environmental factors on disease epidemiology to develop predictive models for better preparedness.

## 5. Contributors to This Special Issue

We extend our deepest gratitude to all the contributors whose invaluable research has made this Special Issue possible. Your dedication and innovative work are instrumental in advancing our collective understanding of Leishmaniasis and bringing us closer to effective solutions.

We hope that this Special Issue will serve as a valuable resource for researchers, healthcare professionals, and policymakers. By fostering continued collaboration and innovation, we can make significant strides towards controlling Leishmaniasis.

Thank you for engaging with this Special Issue. We look forward to witnessing the future breakthroughs in Leishmaniasis research that this collective effort will undoubtedly inspire.

## 6. Concluding Remarks

The research showcased in this Special Issue of Tropical Medicine and Infectious Disease represents significant advances in the field of Leishmaniasis research. The selection of six “Editor’s choice” articles, carefully chosen by the Editorial Board, reflects the outstanding quality and relevance of these contributions. However, it is important to emphasize that all the studies published in this Special Issue have made valuable contributions to our understanding of Leishmaniasis. Each article has its own unique merit, and collectively, they provide a comprehensive overview of current challenges and innovations in the field.

The editorial team would like to express deep appreciation to all authors, reviewers, and especially the readers of Tropical Medicine and Infectious Disease. Your ongoing engagement and support are crucial in driving forward the research and discussion needed to tackle this neglected tropical disease. The insights and progress documented in this Special Issue are important steps toward more effective control, diagnosis, and treatment of Leishmaniasis, and it is through the combined efforts of our global community that such advancements are possible.
